# Fecal Calprotectin as a Biomarker of Crohn's Disease in Patients With Short Disease Durations: A Prospective, Single-Center, Cross-Sectional Study

**DOI:** 10.1155/grp/9984055

**Published:** 2025-04-25

**Authors:** Natsuki Ishida, Shunya Onoue, Tomohiro Takebe, Kenichi Takahashi, Yusuke Asai, Satoshi Tamura, Tomoharu Matsuura, Mihoko Yamade, Moriya Iwaizumi, Yasushi Hamaya, Takanori Yamada, Satoshi Osawa, Ken Sugimoto

**Affiliations:** ^1^First Department of Medicine, Hamamatsu University School of Medicine, Hamamatsu, Shizuoka, Japan; ^2^Department of Laboratory Medicine, Hamamatsu University School of Medicine, Hamamatsu, Shizuoka, Japan; ^3^Department of Endoscopic and Photodynamic Medicine, Hamamatsu University School of Medicine, Hamamatsu, Shizuoka, Japan

**Keywords:** biomarker, C-reactive protein, Crohn's disease, fecal calprotectin, simple endoscopic score for Crohn's disease

## Abstract

**Purpose:** Fecal calprotectin (FC) is a Crohn's disease (CD) biomarker, although the impact of disease duration on its accuracy remains unclear. This study was aimed at investigating the effects of CD disease duration on FC.

**Methods:** In this prospective, single-center, cross-sectional study, we performed 113 endoscopies and biomarker measurements. Endoscopy results were assessed using the simple endoscopic score for Crohn's disease (SES-CD), with an SES-CD ≤ 2 defined as endoscopic remission (ER). Cohort 1 was divided into short-term and long-term disease groups. The associations of the SES-CD with C-reactive protein and FC were analyzed.

**Results:** The correlation coefficient of FC and the SES-CD was 0.670 for all cases. In Cohort 1, the correlation coefficient of FC and the SES-CD was > 0.670 for all subgroups of the short-term disease group (≤ 20 years). The correlation coefficient of FC and CD was < 0.670 for all subgroups of the long-term disease group (> 20 years). In Cohort 2, the correlation coefficients were > 0.670 (0.808) for the 0–4-year disease group and < 0.670 for the 5–14- and 15–40-year disease groups. The receiver-operating characteristic analysis performed to predict ER of all cases resulted in an area under the curve (AUC) of 0.8443, with large AUCs of 0.907, 0.816, and 0.770 observed for the 0–4-, 5–14-, and 15–40-year disease groups, respectively.

**Conclusions:** FC was affected by CD duration, and it may be a useful biomarker of CD, especially in patients with a short disease duration.

## 1. Introduction

Crohn's disease (CD) is a type of inflammatory bowel disease (IBD) and intractable chronic inflammatory disease characterized by recurrent abdominal pain, diarrhea, and bloody stools. CD damages the intestinal tract, resulting in strictures, fistulas, and abscesses that require hospitalization and surgery, and reduces the quality of life of patients [1, 2]. To avoid these poor outcomes, mucosal healing is necessary. Awareness of the clinical symptoms of CD contributes to the reductions in the CD recurrence rate, hospitalization rate, and intestinal resection rate, as clarified in Selecting Therapeutic Targets in Inflammatory Bowel Disease (STRIDE) II [3–5]. Endoscopy is a fundamental evaluation method necessary for mucosal healing. Additionally, the simple endoscopic score for Crohn's disease (SES-CD) is widely applied in clinical practice and large-scale clinical research to evaluate CD [6]. However, frequent endoscopy examinations create physical and mental burdens for patients with CD; therefore, caution should be exercised when managing such patients, including those with preexisting fistulas and strictures. Biomarkers are useful for preventing these burdens and risks; therefore, their use is an intermediate goal of STRIDE II [5].

C-reactive protein (CRP) is a biomarker of CD in clinical practice, and many studies have evaluated its usefulness [7, 8]. Additionally, fecal calprotectin (FC) is a useful biomarker for evaluating the endoscopic activity of CD, and it is superior to CRP for evaluating CD activity [9–12].

Several factors, such as age, obesity, diet, and lack of exercise, affect FC levels [13–15]. We previously reported the effect of disease duration on FC in ulcerative colitis (UC), as well as the improved accuracy of FC and fecal immunochemical occult blood testing for short disease durations [16]. Eder et al. reported an association between disease duration and FC in CD; furthermore, they reported that the correlations of FC with CD durations ≥ 10 years and < 10 years were similar, and that disease duration and time-dependent changes of the CD phenotype did not affect the utility of diagnostic measurements [17]. However, Eder et al. only evaluated 10-year intervals, and no specific disease period has been evaluated in detail [17]. Therefore, we aimed to investigate whether disease duration affects the accuracy of FC and CRP when used as biomarkers for CD. In the present study, the disease duration was grouped more finely, and the relationships between FC and CRP and the endoscopy score were compared between groups to enable more effective biomarker use.

## 2. Materials and Methods

### 2.1. Patients and Study Design

This prospective, single-center, cross-sectional study evaluated 113 measurements of 81 patients with CD who visited Hamamatsu University School of Medicine between February 2020 and July 2023. These patients were diagnosed with CD based on the clinical presentation, endoscopy findings, and histology in accordance with the currently established criteria [18]. Patients with non-CD IBD, such as UC and IBD unclassified, were excluded. Patients with CD who underwent enterostomy were also excluded because their stool sample specimens could not be evaluated accurately. Additionally, patients who used nonsteroidal anti-inflammatory drugs and those with malignancies were excluded because they could affect FC.

The study protocol was reviewed and approved by the ethics committee of Hamamatsu University School of Medicine (number 20-178). This study was conducted in accordance with the principles of good clinical practice according to the Declaration of Helsinki. All the study participants provided written informed consent.

**Figure 1:**
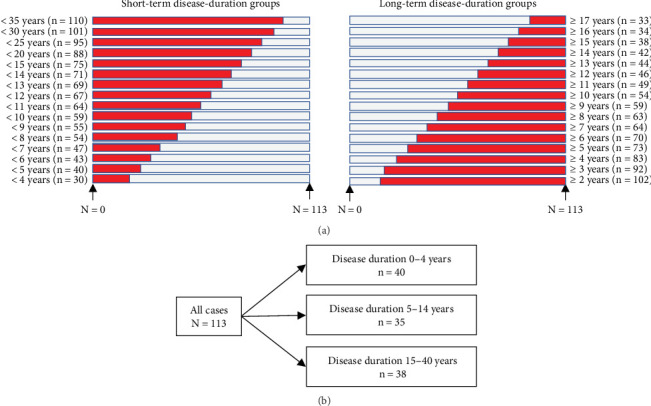
Study flow diagram. (a) Cohort 1: the data of patients with short-term and long-term diseases were extracted and analyzed according to the disease duration. (b) Cohort 2: all cases were divided into three groups based on disease duration so that each group had a similar number of cases.

The effects of disease duration on the correlations between endoscopic activity and FC and CRP were the primary endpoints. The secondary endpoints were endoscopic remission (ER) and its association with disease duration. This study analyzed two groups: Cohort 1 and Cohort 2 (Figure 1). Cohort 1 was created to capture the general trends of the relationships between disease duration and biomarkers. Cohort 2 was created to perform a detailed analysis of the results of Cohort 1. All patients in Cohort 1 were divided into the long-term and short-term groups and analyzed. The disease durations of the groups were adjusted so that each group included a similar number of patients. All patients in Cohort 2 were divided as equally as possible into the following three groups based on disease duration: 0–4 years (*n* = 40), 5–14 years (*n* = 35), and 15–40 years (*n* = 38). The 0–4-, 15–40-, and 5–14-year disease duration groups were analyzed as short-term, long-term, and intermediate disease duration groups, respectively. The correlations and ER of both cohorts were assessed.

### 2.2. Disease Assessment

Clinical activity was assessed using Crohn's disease activity index (CDAI) [19]. Endoscopy evaluations such as colonoscopy and anal double-balloon endoscopy were performed. Before endoscopy was performed, the patients were administered oral polyethylene glycol–based or magnesium citrate–based solutions. All endoscopy procedures were performed by an experienced endoscopist. The SES-CD was used to evaluate the endoscopic score [6]. In the present study, an SES-CD of 0–2 was defined as ER.

### 2.3. Biomarker Measurements

Fecal samples were collected within a few days prior to endoscopy preparation because the solution used to prepare for the examination could affect the FC results. Fecal samples were collected in plastic tubes, and FC measurements were performed; thereafter, the samples were stored at −20°C until they were sent to the laboratory for evaluation (SRL Inc., Tokyo, Japan). Samples were analyzed using a Phadia 250 immunoanalyzer (Hitachi Ltd., Tokyo, Japan) and Elia A Calprotectin 2 reagent (Phadia GmbH, Freiburg, Germany) in accordance with fluorescence enzyme immunoassay principles. CRP levels were measured at our institution as a part of routine clinical practice.

### 2.4. Statistical Analysis

Statistical analyses were performed using IBM SPSS Statistics for Windows Version 24 (IBM Corp., Armonk, NY, United States) and EZR (Saitama Medical Center, Jichi Medical University, Saitama, Japan) software [20]. Correlations between endoscopic scores and biomarkers were assessed using Spearman's rank correlation test. Fisher's exact and the Kruskal–Wallis tests were performed to compare the three groups. A receiver-operating characteristic (ROC) analysis was performed to calculate the cutoff value and area under the curve (AUC) to predict ER. Statistical significance was set at *p* < 0.05.

## 3. Results

### 3.1. Patient Characteristics

This study included the results of 113 endoscopy assessments and biomarker measurements of 81 patients with CD (Table 1). We enrolled 83 male and 30 female patients, with a median age of 40 years and median disease duration of 9 years. Surgery was performed for 33 patients. The median CDAI was 89. The median SES-CD was 3 for both clinical activity and endoscopic activity. The median biomarker values were 0.12 mg/dL and 555 mg/kg for CRP and FC, respectively.

### 3.2. Correlation Between the SES-CD and Biomarkers of Groups With Long-Term and Short-Term Disease

**Figure 2:**
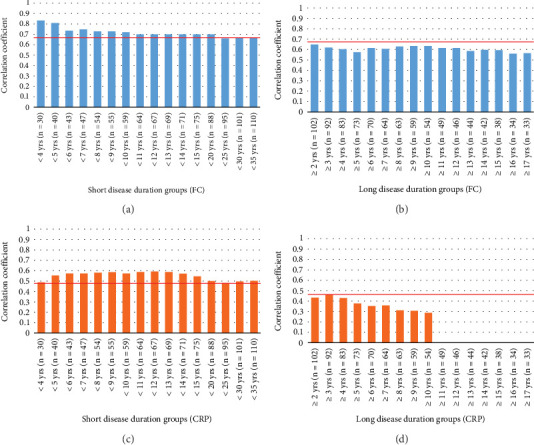
Correlation coefficients of biomarkers and simple endoscopic score for Crohn's disease according to disease duration. Bar graphs of the correlation coefficients of fecal calprotectin (FC) and the simple endoscopic score for Crohn's disease (SES-CD) of the (a) short-term and (b) long-term disease groups. Bar graphs of the correlation coefficients of C-reactive protein (CRP) and the SES-CD of the (c) short-term and (d) long-term disease groups. The solid red line indicates the correlation coefficients of the SES-CD and FC and CRP of all cases.

Correlations of the SES-CD with FC and CRP were analyzed by grouping patients according to their disease duration (short-term and long-term), as performed for Cohort 1 (Figure 2). To ensure that each group had a similar number of patients, the short-term disease group was separated into 1-year subgroups, and the long-term disease group was separated into 5-year subgroups. The correlation coefficient for FC was 0.670 for all cases. A significant correlation between FC and SES-CD was observed in all groups. For the short-term disease group, the correlation coefficient was lower than the overall correlation coefficient of 0.67, which was observed in subgroups with disease durations of 25 (*r* = 0.660), 30 (*r* = 0.667), and 35 (*r* = 0.665) years or longer. The correlation coefficients were > 0.67 for the other disease durations (Figure 2a). The correlation coefficient was < 0.67 for all cases in the long-term disease group (Figure 2b). A similar analysis was performed for CRP, and the correlation coefficient for all cases was 0.458. The correlation coefficient of the short-term disease group was > 0.458 for all cases (Figure 2c). Bar graphs were not presented because a significant correlation was not observed in the long-term disease ≥ 11 years subgroup. The correlation coefficient of the long-term disease group was < 0.458 (Figure 2d). Since the SES-CD includes stenosis, we evaluated the correlation between the score obtained by omitting the stenosis score and FC and CRP to verify the influence of inflammation (Supporting Information 1: Figure S1). The results were similar to those of the aforementioned conventional SES-CD and FC and CRP. Furthermore, the correlation coefficient of the short-term disease group tended to be high; however, that of the long-term disease group tended to be low.

### 3.3. Correlation Between the SES-CD and Biomarkers and ROC Analysis Performed to Predict ER of the Three Disease Duration Groups

The baseline characteristics of patients in Cohort 2 are presented in Table 1. These patients were separated into three groups according to the disease duration. Significant differences in age, sex, Montreal criterion B, history of surgery, and immunomodulator use were observed (*p* < 0.001, *p* = 0.012, *p* < 0.001, *p* < 0.001, and *p* = 0.003, respectively). We analyzed the correlations between the SES-CD, FC, and CRP among the three groups (Table 2). The correlation coefficient was 0.808 for the 0–4-year disease group, which was higher than the overall correlation coefficient of 0.670; however, the correlation coefficients of the 5–14- and 15–40-year disease groups were < 0.670. The CRP values were higher than the overall correlation coefficient of 0.458 for the 0–4- and 5–14-year disease groups; however, no significant correlation was observed in the 15–40-year disease group. The ROC analysis performed to predict ER for all cases indicated cutoff values of 424 mg/kg for FC and 0.15 mg/dL for CRP, with AUCs of 0.843 (95% confidence interval (CI): 0.769–0.918) and 0.715 (95% CI: 0.620–0.810), respectively (Table 3). Similarly, the AUC of FC of the 0–4-year disease group was larger than that of the entire group and smaller than that of the 5–14- and 15–40-year disease groups; however, no metric difference was observed. The AUCs of CRP of the 0–4- and 5–14-year disease groups were larger than that of the entire group; however, no metric difference was observed.

### 3.4. Association of the Disease Duration With Age, Sex, Montreal Criterion B, History of Surgery, and Immunomodulator Use

**Figure 3:**
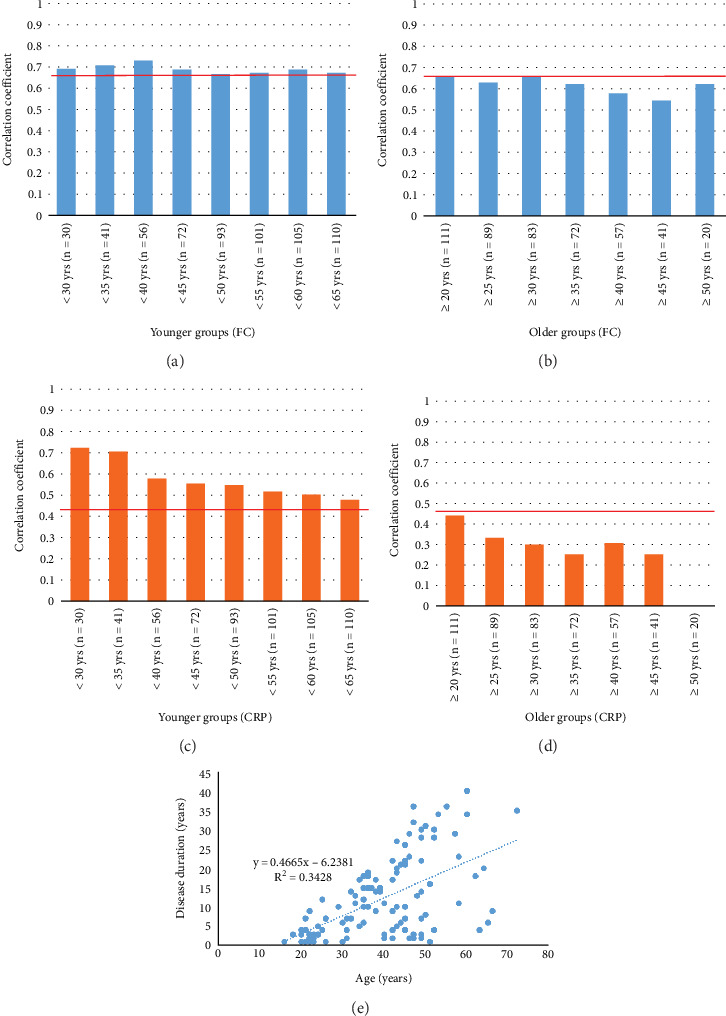
Correlation coefficients of biomarkers and the simple endoscopic score for Crohn's disease according to age. Bar graphs of the correlation coefficients of fecal calprotectin (FC) and the simple endoscopic score for Crohn's disease (SES-CD) of the (a) younger and (b) older age groups. Bar graphs of the correlation coefficients of C-reactive protein (CRP) and the SES-CD of the (c) younger and (d) older age groups. The solid red line indicates the correlation coefficients of the SES-CD and FC and CRP for all cases. (e) Scatter plots of the correlations between disease duration and age.

A comparison of the baseline characteristics of the three groups revealed significant differences in age, sex, Montreal criterion B, history of surgery, and immunomodulator use (Table 1). Since these factors may be associated with disease duration and affect biomarkers, we assessed the association of each item with the ability of the biomarkers to accurately predict CD. First, we evaluated the relationship between age and the biomarkers. After considering the age and distribution of the patients, the groups were divided using 5-year intervals. Regarding the correlation between FC and the SES-CD of the younger group, the correlation coefficient was > 0.670, but not for patients younger than 50 years (Figure 3a). In the older group, the correlation coefficient of FC and SES-CD was < 0.670 (Figure 3b). The correlation coefficients of CRP and SES-CD of the younger and older groups were > 0.458 and < 0.458, respectively (Figure 3c,d). Age showed the same tendency as disease duration, and disease duration was significantly correlated with age (Figure 3e). Age and disease duration were associated with CD, and both had similar effects on the correlations between the SES-CD and FC and CRP.

Additionally, we assessed sex, Montreal criterion B, history of surgery, and immunomodulator use, which were categorical variables. However, age was a continuous variable. Therefore, the correlation between the SES-CD and biomarkers was evaluated, and the patients were divided into two groups: short-term and long-term disease. As a result, the correlation coefficients of each variable were distributed as evenly as possible (Table 4). The correlation coefficients (except for those of the Montreal criterion B3 and immunomodulator use subgroups) of the SES-CD and FC of the short-term disease group were higher than those of the long-term disease group.

## 4. Discussion

Our study showed that FC may be superior to CRP for reflecting endoscopic activity of short-term disease. Additionally, a comparison of the three groups in Cohort 2 indicated that age, sex, Montreal criterion B, history of surgery, and immunomodulator use may affect disease duration. Although age was associated with disease duration, other factors had greater correlations with disease duration in the short-term disease group, indicating that disease duration affected FC. Mucosal healing and transmural healing are the goals of IBD treatment, and biomarkers are important for establishing treatment guidelines for IBD [5]. Although FC is a biomarker that accurately reflects the mucosal status of IBD, it does not completely reflect it because of variables such as age, obesity, diet, and lack of exercise [13–15]. Sampling is an external factor. Calafat et al. reported that the accuracy of FC cannot be guaranteed, particularly for patients with active UC [21]. Diarrheal stools are common among patients with active UC, and poor sampling is often observed in daily clinical practice. Kolho et al. reported the effects of laxatives and discouraged obtaining stool samples during the oral administration of the bowel preparation solution before endoscopy [22].

As mentioned, various factors can affect FC. We previously reported that disease duration can affect FC in UC [16]. We performed a similar analysis during our previous study that evaluated the association between FC and fecal immunochemical blood test results and disease duration of UC [16]. Additionally, in our previous study, we analyzed the endoscopic score using the Mayo endoscopic subscore, UC Endoscopic Index of Severity, and the sum of the Mayo endoscopic subscore [16]. However, in the present study, Cohort 2, which was divided into three groups according to disease duration, exhibited significant differences in age, sex, Montreal criterion B, history of surgery, and immunomodulator use. Therefore, it was necessary to determine whether these factors affect FC. Since age is a continuous variable, analyses of the younger and older groups were performed separately, similar to the analysis of the disease duration of Cohort 1. The correlation coefficients of the younger group tended to be higher than those of the older group. There was a significant correlation between disease duration and age (Figure 3e), suggesting that these factors also affect the accuracy of FC. Although Li et al. reported a relationship between age and FC, their study focused on children 1–18 months of age [13]. To the best of our knowledge, this is the first study to examine the effects of disease duration and age on FC in adults. Regarding sex, Montreal criterion B, history of surgery, and immunomodulator use, the correlation coefficients of the short-term disease group (except for those of the Montreal criterion B3 and immunomodulator use subgroups) were higher than those of the long-term disease group. In studies where the analysis included SES-CD for CD, there was a concern that surgical history may affect the endoscopic score; however, notably, the correlation coefficient for FC was higher in patients with a shorter disease duration, regardless of surgical history. Regarding the Montreal criterion B3 and immunomodulator use subgroups, their smaller sample sizes (compared with those of other subgroups) may have affected the results; therefore, it is necessary to evaluate more cases. Even when factors such as age, sex, Montreal criterion B, history of surgery, and immunomodulator use were considered, the accuracy of FC tended to be a relatively better biomarker of short-term disease. As a precaution, we used linear regression analysis to evaluate the relationships between disease duration and FC and CRP (Supporting Information 2: Table S1). Our results showed no significant association between disease duration and FC (*β* = 0.52, 95% CI: −50.81 to 51.86, *p* = 0.98). Although there was no direct association between FC and disease duration, considering the results of the previous analysis, an association between FC and disease duration may be shown with the inclusion of the endoscopic score. Eder et al. studied the effect of disease duration on FC in CD and concluded that disease duration and time-dependent changes in the CD phenotype did not affect the diagnostic utility of FC measurements [17]. However, during the analysis by Eder et al., disease duration was divided into only two groups (≥ 10 years and < 10 years), and the lack of more detailed groups seemed to be a limitation. The reason why FC was more accurate in the short-term disease group is unknown; however, as discussed in our previous study, we hypothesized that neutrophils are less likely to be present because of intestinal fibrosis progression and inflammation-associated oxidative stress, which might damage mucosal DNA, thus altering the profile of inflammatory cytokines in the gut and affecting FC, which reflects neutrophils in the gut, as calprotectin is a neutrophilic protein [16, 23].

This study had some limitations. First, we analyzed a small number of target patients at a single institution, and the sample size was further reduced by subgrouping. The small sample size made multiple adjustments difficult. In Cohort 1, the number of cases in each group was different; therefore, it was not possible to evaluate an equal number of patients. In the long-term disease group, the number of patients decreased correspondingly with the increased number of years. Conversely, an analysis of the short-term group showed that the correlation coefficient was particularly high until approximately 10 years, regardless of the decrease in the number of people, thus supporting the high correlation coefficient of the short-term disease group. Regarding Cohort 2, it was possible to separate the patients into only three groups because of the small sample size; however, it is necessary to increase the number of groups to determine which period would particularly benefit from the use of biomarkers. In addition, the relatively mild endoscopic disease activity in all patient groups may have affected the ability to identify the correlation of FC with endoscopic disease activity. Furthermore, in the present study, disease duration was defined as the time from the diagnosis. However, CD onset may have occurred earlier. No comparisons with leucine-rich alpha-2 glycoprotein, which is also a useful biomarker, were performed [24–27]. Additionally, multiple endoscopies and measurements of each patient were necessary to ensure an adequate sample size.

## 5. Conclusions

FC in CD is affected by the disease duration. Therefore, FC may be a more useful biomarker of CD with shorter disease durations.

## Figures and Tables

**Table 1:** Comparison of baseline characteristics of the three groups according to disease duration.

**Characteristics**	**All patients**	**Disease duration, 0–4 years**	**Disease duration, 5–14 years**	**Disease duration, 15–40 years**	**p** ** value**
	**N** = 113	**n** = 40	**n** = 35	**n** = 38	
Age (year), median [IQR]	40 [26, 47]	23 [21, 42]	36 [31, 44]	46 [42, 52]	< 0.001
Male/female, *n* (%)	83 (73.5)/30 (26.5)	24 (60.0)/16 (40.0)	25 (71.4)/10 (28.6)	34 (89.5)/4 (10.5)	0.012
Disease duration (year), median [IQR]	9 [3, 18]	2 [1, 3]	9 [7, 11]	22 [18, 29]	< 0.001
Montreal criteria					
A1/A2/A3, *n* (%)	7 (6.2)/94 (83.2)/12 (10.6)	3 (7.5)/30 (75.0)/7 (17.5)	3 (8.6)/29 (82.9)/3 (8.6)	1 (2.6)/35 (92.1)/2 (5.3)	0.304
L1/L2/L3, *n* (%)	37 (32.7)/12 (10.6)/64 (56.6)	15 (37.5)/4 (10.0)/21 (52.5)	7 (20.0)/4 (11.4)/24 (68.6)	15 (39.5)/4 (10.5)/19 (50.0)	0.424
B1/B2/B3, *n* (%)	39 (34.5)/37 (32.7)/37 (32.7)	22 (55.0)/14 (35.0)/4 (10.0)	15 (42.9)/6 (17.1)/14 (40.0)	2 (5.3)/17 (44.7)/19 (50.0)	< 0.001
Surgery, *n* (%)	33 (29.2)	3 (7.5)	11 (31.4)	19 (50.0)	< 0.001
CDAI, median [IQR]	89 [37, 143]	58 [24, 122]	83 [44, 146]	105 [63, 146]	0.052
CRP (mg/dL), median [IQR]	0.12 [0.04, 0.25]	0.12 [0.04, 0.39]	0.12 [0.04, 0.23]	0.11 [0.05, 0.18]	0.842
FC (mg/kg), median [IQR]	555 [149, 1970]	743 [219, 3772]	696 [109, 2100]	263 [111, 1005]	0.087
SES-CD	3 [2, 5]	3 [2, 6]	3 [1, 5]	3 [1, 4]	0.366
Medication during the study, *n* (%)					
Oral 5-ASA	103 (91.2)	35 (87.5)	32 (91.4)	36 (94.7)	0.530
Systemic steroids	7 (6.2)	3 (7.5)	2 (5.7)	2 (5.3)	0.910
Immunomodulators	30 (26.5)	3 (7.5)	13 (37.1)	14 (36.8)	0.003
Biologics	83 (73.5)	26 (65.0)	30 (85.7)	27 (71.1)	0.118
ED	49 (43.4)	21 (52.5)	12 (34.3)	16 (42.1)	0.278

Abbreviations: 5-ASA, 5-aminosalicylic acid; CDAI, Crohn's disease activity index; CRP, C-reactive protein; ED, elemental diet; FC, fecal calprotectin; IQR, interquartile range; SES-CD, simple endoscopic score for Crohn's disease.

**Table 2:** Correlation coefficients of the simple endoscopic score for Crohn's disease and FC or CRP.

	**All**		**Disease duration, 0–4 years**		**Disease duration, 5–14 years**		**Disease duration, 15–40 years**	
	**N** = 113		**n** = 40		**n** = 35		**n** = 38	
	**r**	**p**	**r**	**p**	**r**	**p**	**r**	**p**
FC	0.670	< 0.001	0.808	< 0.001	0.552	< 0.001	0.599	< 0.001
CRP	0.458	< 0.001	0.554	< 0.001	0.538	< 0.001	0.176	0.290

Abbreviations: CRP, C-reactive protein; FC, fecal calprotectin.

**Table 3:** Receiver-operating characteristic analysis performed to predict a simple endoscopic score of 0–2 for Crohn's disease.

		**Cutoff value**	**Sensitivity**	**Specificity**	**PPV**	**NPV**	**Accuracy**	**AUC [95% CI]**	**p** ** value**
All	FC	424	0.779	0.882	0.869	0.712	0.796	0.843 [0.769–0.918]	0.028
*N* = 113	CRP	0.15	0.544	0.800	0.804	0.537	0.646	0.715 [0.620–0.810]	

Disease duration, 0–4 years	FC	452	0.846	0.786	0.880	0.733	0.825	0.907 [0.816–0.997]	0.167
*n* = 40	CRP	0.07	0.808	0.786	0.875	0.688	0.800	0.791 [0.643–0.939]	

Disease duration, 5–14 years	FC	483	0.857	0.786	0.857	0.786	0.829	0.816 [0.656–0.976]	0.677
*n* = 35	CRP	0.07	0.857	0.571	0.750	0.727	0.743	0.765 [0.600–0.931]	

Disease duration, 15–40 years	FC	276	0.824	0.714	0.833	0.700	0.763	0.770 [0.614–0.927]	0.021
*n* = 38	CRP	0.11	0.571	0.588	0.500	0.389	0.447	0.464 [0.271–0.656]	

Abbreviations: 95% CI, 95% confidence interval; AUC, area under the curve; CRP, C-reactive protein; FC, fecal calprotectin; NPV, negative predictive value; PPV, positive predictive value.

**Table 4:** Comparison of correlation coefficients of the short-term and long-term disease groups according to sex, Montreal criterion B, history of surgery, and immunomodulator use.

		**Disease duration**	**n**	**FC**		**CRP**	
				**r**	**p**	**r**	**p**
Sex	Male	All	83	0.605	< 0.001	0.428	< 0.001
		Short (≤ 10 years)	41	0.590	< 0.001	0.658	< 0.001
		Long (≥ 11 years)	42	0.580	< 0.001	0.056	0.723
	Female	All	30	0.852	< 0.001	0.550	0.0016
		Short (≤ 3 years)	14	0.856	< 0.001	0.330	0.249
		Long (≥ 4 years)	16	0.825	< 0.001	0.770	< 0.001

Montreal criterion B	B1	All	39	0.794	< 0.001	0.607	< 0.001
		Short (≤ 3 years)	18	0.860	< 0.001	0.503	0.033
		Long (≥ 4 years)	21	0.753	< 0.001	0.716	< 0.001
	B2	All	37	0.710	< 0.001	0.365	0.026
		Short (≤ 10 years)	19	0.733	< 0.001	0.598	0.007
		Long (≥ 11 years)	18	0.716	< 0.001	0.012	0.961
	B3	All	37	0.387	0.018	0.309	0.063
		Short (≤ 10 years)	18	0.323	0.164	0.324	0.163
		Long (≥ 11 years)	19	0.367	0.148	0.160	0.538

Surgery	No surgery	All	80	0.717	< 0.001	0.620	< 0.001
		Short (≤ 5 years)	39	0.766	< 0.001	0.649	< 0.001
		Long (≥ 6 years)	41	0.632	< 0.001	0.572	< 0.001
	Surgery	All	33	0.575	< 0.001	−0.110	0.541
		Short (≤ 15 years)	17	0.619	0.008	−0.260	0.314
		Long (≥ 16 years)	16	0.554	0.026	−0.260	0.314

Immunomodulator use	No immunomodulator use	All	83	0.779	< 0.001	0.450	< 0.001
		Short (≤ 6 years)	43	0.861	< 0.001	0.560	< 0.001
		Long (≥ 7 years)	40	0.711	< 0.001	0.307	0.054
	Immunomodulator use	All	30	0.310	0.095	0.492	0.006
		Short (≤ 13 years)	16	−0.077	0.776	0.504	0.047
		Long (≥ 14 years)	14	−0.115	0.696	0.382	0.178

Abbreviations: CRP, C-reactive protein; FC, fecal calprotectin.

## Data Availability

All data required to evaluate the conclusions of this study are presented herein. Additional data related to this study can be obtained upon request from the authors.

## Supporting Information

Supporting Information 1 Figure S1: Correlation coefficients of biomarkers and the simple endoscopic score for Crohn's disease (SES-CD) without narrowing scores grouped according to disease duration. Bar graphs of the correlation coefficients of fecal calprotectin (FC) and the SES-CD without narrowing scores of the short-term (a) and long-term (b) disease groups. Bar graphs of the correlation coefficients of C-reactive protein (CRP) and the SES-CD without narrowing scores of the short-term (c) and long-term (d) disease groups. The solid red line indicates the correlation coefficients of the SES-CD and FC and CRP without narrowing scores of all cases.

Supporting Information 2 Table S1: Multivariate linear regression analysis of each biomarker.
