# Supplementary material for: Fecal Calprotectin as a Biomarker of Crohn's Disease in Patients With Short Disease Durations: A Prospective, Single-Center, Cross-Sectional Study
Source: Gastroenterol Res Pract. 2025 Apr 25;2025:9984055. doi: 10.1155/grp/9984055 (PMC12048189; doi:10.1155/grp/9984055)
Supplement: Supporting Information 1 — Figure S1: Correlation coefficients of biomarkers and the simple endoscopic score for Crohn's disease (SES-CD) without narrowing scores grouped according to disease duration. Bar graphs of the correlation coefficients of fecal calprotectin (FC) and the SES-CD without narrowing scores of the short-term (a) and long-term (b) disease groups. Bar graphs of the correlation coefficients of C-reactive protein (CRP) and the SES-CD without narrowing scores of the short-term (c) and long-term (d) disease groups. The solid red line indicates the correlation coefficients of the SES-CD and FC and CRP without narrowing scores of all cases. [file 9984055.f1.pptx]

## Slide 1
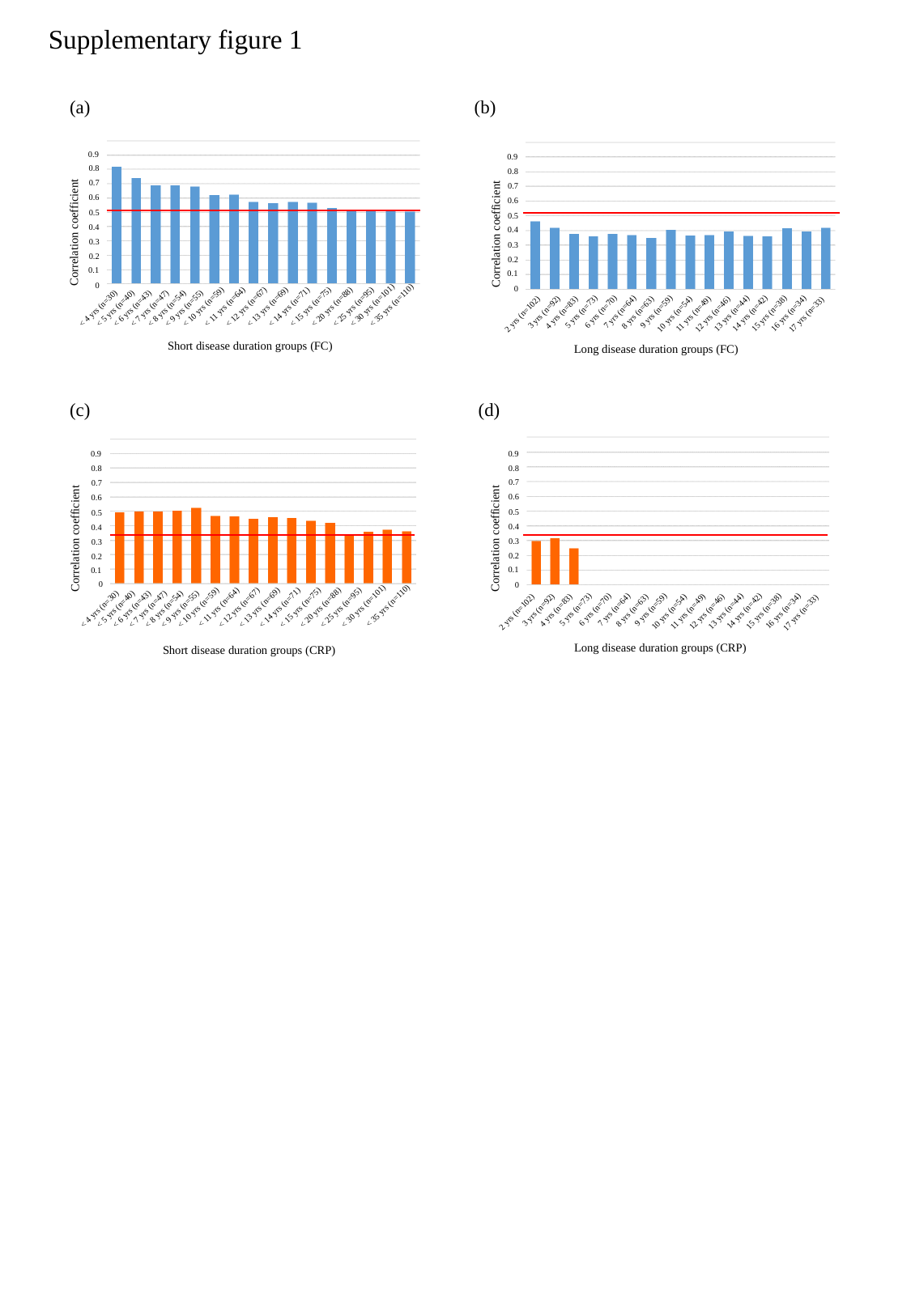

Supplementary figure 1
(a)
(b)
1
1
0.9
0.9
0.8
0.8
0.7
0.7
0.6
0.6
0.5
0.5
0.4
0.4
Correlation coefficient
Correlation coefficient
0.3
0.3
0.2
0.2
0.1
0.1
0
0
< 30 yrs (n=101)
< 35 yrs (n=110)
< 10 yrs (n=59)
< 11 yrs (n=64)
< 12 yrs (n=67)
< 13 yrs (n=69)
< 14 yrs (n=71)
< 15 yrs (n=75)
< 20 yrs (n=88)
< 25 yrs (n=95)
< 4 yrs (n=30)
< 5 yrs (n=40)
< 6 yrs (n=43)
< 7 yrs (n=47)
< 8 yrs (n=54)
< 9 yrs (n=55)
Short disease duration groups (FC)
Long disease duration groups (FC)
(c)
(d)
1
1
0.9
0.9
0.8
0.8
0.7
0.7
0.6
0.6
0.5
0.5
0.4
0.4
Correlation coefficient
Correlation coefficient
0.3
0.3
0.2
0.2
0.1
0.1
0
0
< 30 yrs (n=101)
< 35 yrs (n=110)
< 10 yrs (n=59)
< 11 yrs (n=64)
< 12 yrs (n=67)
< 13 yrs (n=69)
< 14 yrs (n=71)
< 15 yrs (n=75)
< 20 yrs (n=88)
< 25 yrs (n=95)
< 4 yrs (n=30)
< 5 yrs (n=40)
< 6 yrs (n=43)
< 7 yrs (n=47)
< 8 yrs (n=54)
< 9 yrs (n=55)
Long disease duration groups (CRP)
Short disease duration groups (CRP)
