# Supplementary material for: Fecal Calprotectin as a Biomarker of Crohn's Disease in Patients With Short Disease Durations: A Prospective, Single-Center, Cross-Sectional Study
Source: Gastroenterol Res Pract. 2025 Apr 25;2025:9984055. doi: 10.1155/grp/9984055 (PMC12048189; doi:10.1155/grp/9984055)
Supplement: Supporting Information 2 — Table S1: Multivariate linear regression analysis of each biomarker. [file 9984055.f2.docx]

**Supplementary Table 1. Multivariate linear regression analysis of each biomarker**

| Response variables | FC | | | | | CRP | | | | |
| --- | --- | --- | --- | --- | --- | --- | --- | --- | --- | --- |
| Variables | β | 95% CI | SE | t-value | P-value | β | 95% CI | SE | t-value | P-value |
| Intercept | 3073 | 1654 - 4492 | 715.6 | 4.290 | <0.001 | 0.404 | 0.007 - 0.800 | 0.200 | 2.019 | 0.046 |
| Disease duration | 0.520 | -50.81 - 51.86 | 25.90 | 0.020 | 0.980 | -0.002 | -0.017 - 0.012 | 0.007 | -0.314 | 0.754 |
| Male sex | -321.6 | -1329- 685.9 | 508.2 | -0.630 | 0.530 | -0.011 | -0.293 - 0.270 | 0.142 | -0.079 | 0.937 |
| Surgery | 124.5 | -862.0 - 1111 | 497.6 | 0.250 | 0.800 | 0.087 | -0.188 - 0.363 | 0.139 | 0.629 | 0.531 |
| Age | -33.79 | -74.54 - 6.960 | 20.56 | -1.640 | 0.100 | -0.002 | -0.014 - 0.009 | 0.006 | -0.360 | 0.719 |
| Immunomodulator | -225.8 | -1222 – 770.4 | 502.6 | -0.450 | 0.650 | -0.113 | -0.392 - 0.165 | 0.140 | -0.807 | 0.422 |

FC, fecal calprotectin; β, Regression coefficient; CRP, C-reactive protein; β, regression coefficient; CI, confidence interval; SE, standard error.
